# Study on the Oxidation Behavior of TiB_2_-CeO_2_-Modified (Nb,Mo,Cr,W)Si_2_ Coating on the Surface of Niobium Alloy

**DOI:** 10.3390/ma17215244

**Published:** 2024-10-28

**Authors:** Xiaojun Zhou, Lairong Xiao, Yitao Zha, Jiawei Xu, Jiashu Fang, Guanzhi Deng, Shaofu Xu, Sainan Liu, Xiaojun Zhao, Zhenyang Cai

**Affiliations:** 1 School of Materials Science and Engineering, Central South University, Changsha 410083, China; zxj745301@126.com (X.Z.); xiaolr@csu.edu.cn (L.X.); 233111018@csu.edu.cn (Y.Z.); xpluto1211@163.com (J.X.); 223111043@csu.edu.cn (G.D.); 19911765662@163.com (S.X.); zhaoxj@csu.edu.cn (X.Z.); 2 State Key Laboratory of Special Rare Metal Materials, Northwest Rare Metal Materials Research Institute, Shizuishan 753000, China; 3 Powder Metallurgy Research Institute, Central South University, Changsha 410083, China; 8204212428@csu.edu.cn; 4 School of Minerals Processing and Bioengineering, Central South University, Changsha 410083, China; lsn@csu.edu.cn

**Keywords:** TiB_2_, composite coating, static oxidation life, MoSi_2_, CrSi_2_, thermal shock cycle

## Abstract

A novel TiB_2_-CeO_2_-modified (Nb,Mo,Cr,W)Si_2_ coating was prepared on a Nb-5W-2Mo-1Zr alloy substrate using two-step slurry sintering and halide-activated pack cementation to address the limitations of a single NbSi_2_ coating in meeting the service requirements of niobium alloys at elevated temperatures. At 1700 °C, the static oxidation life of the coating exceeded 20 h, thus indicating excellent high-temperature oxidation resistance. This was due to the formation of a TiO_2_-SiO_2_-Cr_2_O_3_ composite oxide film on the coating surface, which, due to low oxygen permeability, effectively prevented the inward infiltration of oxygen. Additionally, the dense structure of the composite coating further enhanced this protective effect. The composite coating was able to withstand over 1600 thermal shock cycles from room temperature to 1700 °C, and its excellent thermal shock performance could be attributed to the formation of MoSi_2_, CrSi_2_, and WSi_2_ from elements such as Mo, Cr, and W, which were added during modification. In addition to adjusting the difference in thermal expansion coefficients between the layers of composite coatings to reduce the thermal stress generated by thermal shock cycles, the formation of silicide compounds also improved the overall fracture toughness of the coating and thereby improved its thermal shock resistance.

## 1. Introduction

Nickel-based high-temperature alloys with a maximum working temperature of around 1300 °C are typically used in traditional aerospace aircraft engines and engine blades as structural materials. However, the current service temperature of new-generation aircraft and other components in aerospace engineering is much higher than 1300 °C and can reach as high as 1600–1800 °C. This necessitates the use of new materials with better high-temperature mechanical properties [1]. Nb-based alloys are high-temperature structural materials that are commonly used in the new generation of aerospace applications because of their high melting point (2468 °C), low density (8.57 g/cm^3^), good processing performance due to enhanced plasticity, and excellent high-temperature mechanical properties [2,3,4]. However, the poor resistance of Nb to oxidation at high temperatures results in severe oxidation above 600 °C. The oxidation product Nb_2_O_5_ is loose and porous, which can cause the rapid oxidation of the niobium alloy substrate due to its ineffectiveness in preventing the inward diffusion of oxygen. This is a serious disadvantage that restricts the further application of niobium alloys in the field of high-temperature applications [5].

Various approaches have been investigated to enhance the high-temperature oxidation resistance of niobium alloys, among which the three main routes are the alloying [6], grain refinement, and surface protective coating methods [7]. The alloying method refers to the addition of Si, Al, Cr, Ti, Hf, and various rare-earth elements to Nb substrates [8], thus forming a dense oxide film by reacting with oxygen and thereby protecting the substrate. The grain refinement method can accelerate the preferential oxidation of alloying elements and is generally used for niobium alloys after alloying. These two methods are able to improve the high-temperature oxidation resistance of niobium alloys to a certain extent; however, the substantial addition of antioxidant alloy elements often leads to rapid deterioration of the mechanical properties of niobium alloys. Consequently, balancing processing performance with complete oxidation resistance in high-temperature environments above 1300 °C remains challenging, thus limiting the application potential of the existing niobium alloys [9]. In the surface protective coating method, a protective barrier is prepared on the surface of the niobium alloy substrate, which can protect the substrate at high temperatures by effectively preventing the inward diffusion of oxygen. Presently, the various high-temperature oxidation-resistant coatings for niobium alloys include precious metal coatings, oxide ceramic coatings, heat-resistant alloy coatings, aluminide coatings, silicide coatings, etc. [10,11,12,13,14,15,16], among which silicide coatings are the most widely used. Due to the semi-molten state of SiO_2_ and its fluidity at high temperatures, it can repair coating cracks by flowing into the coating, thus endowing certain self-healing properties [17,18]. This coating system is significant in the high-temperature protection of niobium alloys.

Among the various silicide coating systems that are similar to niobium alloys are Si-Cr-Ti(Fe) coatings [19], Si-Nb coatings [20,21], and Si-Mo coatings [22,23]. Presently, the Si-Nb coating is the most widely used, with NbSi_2_ as the main phase of the coating. However, the service requirements at higher temperatures cannot be met by single NbSi_2_ coatings. Additionally, the mismatch in the thermal expansion coefficient between the coating and the substrate can easily cause defects, such as cracks, which can ultimately lead to failure in high-temperature thermal shock environments. Therefore, doping the NbSi_2_ coating is necessary [24,25,26] for the preparation of composite coatings with excellent high-temperature protection abilities and resistance to cyclic thermal shock. TiB_2_ and MoSi_2_ both have high melting points and excellent high-temperature oxidation resistance [22,27]. CeO_2_ can promote the thickness of the NbSi_2_-MoSi_2_ coating and improve grain refinement [2]. Yue et al. [28] have prepared a MoSi_2_/WSi_2_ compound coating on a Nb-Ti-Si-based alloy, in which WSi_2_ has a Si storage function, thereby improving the service life of the coating. Zhang [29] has prepared a MoSi_2_-CrSi_2_-Si_3_N_4_ composite coating on a Mo substrate and found that the Cr element inhibits the diffusion of Si during oxidation and the formation of a volatilizable MoO_3_ phase. The mismatch in the thermal expansion coefficient between NbSi_2_ and niobium alloy substrates can be reduced by introducing CeO_2_, TiB_2_, MoSi_2_, CrSi_2_, and WSi_2_.

Therefore, in this study, a TiB_2_-CeO_2_-modified (Nb,Mo,Cr,W)Si_2_ coating was prepared on a Nb-5W-2Mo-1Zr alloy substrate using two-step slurry sintering and halide-activated pack cementation. The formation mechanism, static oxidation resistance, thermal shock resistance, and microstructural properties of the coating at 1700 °C were studied.

## 2. Experimental Procedure

### 2.1. Nb Alloy Substrate Preparation

Nb521 alloy (Nb-5W-2Mo-1Zr, wt.%, Ningxia Orient Tantalum Industry Co., Ltd., Ningxia, China) was used as the substrate in this study. Firstly, 70 mm × 7.5 mm × 0.8 mm niobium sheets were cut from the substrate, and a hole with a diameter of 3 mm was drilled at one end of each niobium sheet for hanging in the sintering furnace. Each specimen of the niobium sheet was grounded and polished, followed by ultrasonication in NaOH solution at 100 °C for 10 min (pH = 13) for the removal of oil. Each specimen was then cleaned in distilled water and subsequently ultrasonically cleaned for 10 min at 80–90 °C in a mixed solution of HNO_3_, HCl, and deionized water at a pH value of approximately 1 to remove the oxide layer from the sample surface. Ultrasonic cleaning with alcohol was used for final cleaning of the Nb521 specimens.

### 2.2. Preparation of the Coating

**Figure 1:**
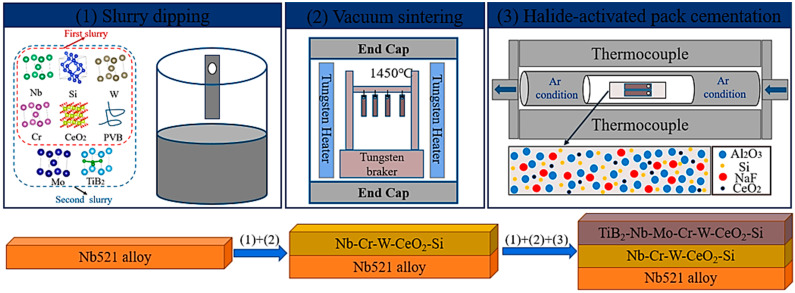
Schematic diagram of the preparation process of coating.

The preparation process of the TiB_2_-CeO_2_-modified (Nb,Mo,Cr,W)Si_2_ coating is shown in Figure 1. Two-step slurry sintering and halide-activated pack cementation were used to prepare the TiB_2_-CeO_2_-modified (Nb,Mo,Cr,W)Si_2_ coating on the Nb521 substrate. Nb-Cr-W-Si and TiB_2_-Nb-Mo-Cr-W-Si were the main solutes of the slurries used for the two dippings, and anhydrous ethanol was used as the solvent. In addition, during the sintering process, PVB and extremely trace amounts of CeO_2_ (to improve the uniformity of the coating structure) were also added. The purity of all of the powders was over 99.9%, and all of the reagents were of AR grade. The ratio of solute (g) to ethanol solvent (mL) was selected as 1:0.9. The slurry was placed in a planetary mill (ZrO_2_ as the ball milling medium) before use and fully grounded for 8 h at 250 rpm. In order to ensure that the surface of the slurry on the substrate was uniform and that the thickness was moderate, each slurry dipping was conducted in three intervals, with each lasting 5–8 s. After each dipping, the Nb521 substrates were dried at 60 °C for 12 h and sintered in a vacuum sintering furnace (1 × 10^−3^ Pa) at 1450 °C for 120 min after heating at a rate of 10 °C/min. The two layers of the coating formed by the two dippings and by the sintering were CeO_2_-Nb-Cr-W-Si and CeO_2_-TiB_2_-Nb-Mo-Cr-W-Si, respectively. After two-step slurry sintering, the sample was embedded into a uniform mixture of Al_2_O_3_ (purity 99.9%), NaF powder (purity 99.9%), CeO_2_ powder, and Si (purity 99.9%) and maintained at 1200 °C in an argon-protected tube furnace in order to facilitate the formation of silicide components in the surface coating of the sample.

### 2.3. Characterization of Microstructure

Phases on the coating were analyzed using X-ray diffraction (XRD, D/Max 2500, Rigaku Corporation, Tokyo, Japan) employing a Cu Kα radiation of 30 kV and 100 mA. For morphological evaluation of the coating and performance of qualitative and semi-quantitative analysis on the surface and cross-section of the coating, we employed a scanning electron microscope (SEM, sirion200, FEI Company, Hillsboro, OR, USA) equipped with an energy dispersive spectrometer (EDS). Electron probe microanalysis (EPMA, JXA-8230, Japan Electronics Co., Ltd., Tokyo, Japan) equipped with wavelength dispersive spectroscopy (WDS) was used to analyze the morphology of coating cross-sections and quantitative analysis of the composition. High-resolution transmission electron microscopy (HRTEM, Titan G2 60-300, FEI Company, Hillsboro, OR, USA) was used to characterize the crystal structure of the main coating phase.

### 2.4. Oxidation Resistance and Thermal Shock Resistance Tests of the Coating

A self-developed high-temperature oxidation experimental setup was employed to carry out the static oxidation resistance test and high-temperature thermal shock resistance test of the coating. In the static antioxidant test, the coating on both ends of the specimen was polished off and clamped to introduce a high-current circuit. Subsequently, the specimen was heated to the oxidation temperature (1700 °C) at a rate of 600 °C/min using the Joule effect and maintained at this temperature for the entire duration of the test. For the high-temperature thermal shock resistance test of the coating, one thermal shock cycle consisted of heating the sample from room temperature to 1700 °C within 15 s (the heating method used during the high-temperature thermal shock resistance test was the same as that used during the static oxidation resistance test), holding the temperature for 10 s, and then cooling from 1700 °C to room temperature within 10 s using cooling water. This was followed by holding at room temperature for 5 s before reheating. The number of thermal shock resistance cycles of the coating was recorded automatically by the system. Both the static oxidation test and thermal shock test were carried out in a static air environment.

## 3. Result and Discussion

### 3.1. Surface Morphology of the Original Coating

**Figure 2:**
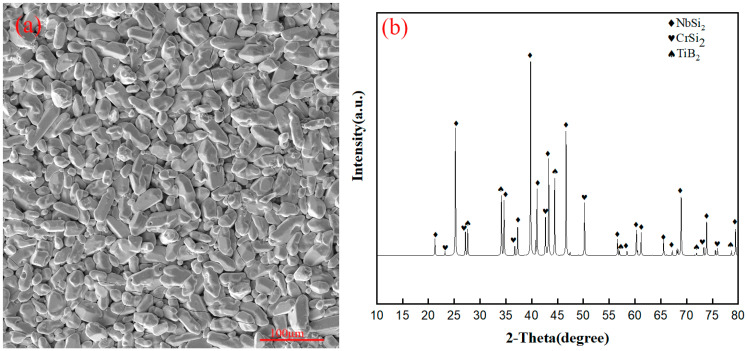
(**a**) Surface morphology of the original coating and its (**b**) XRD spectrum.

The surface morphology of the original coating, along with its XRD analysis results, is shown in Figure 2. The surface composition of the original coating can be seen to contain NbSi_2_, CrSi_2_, and TiB_2_, with the overall composition being uniform with only a few microcracks.

**Figure 3:**
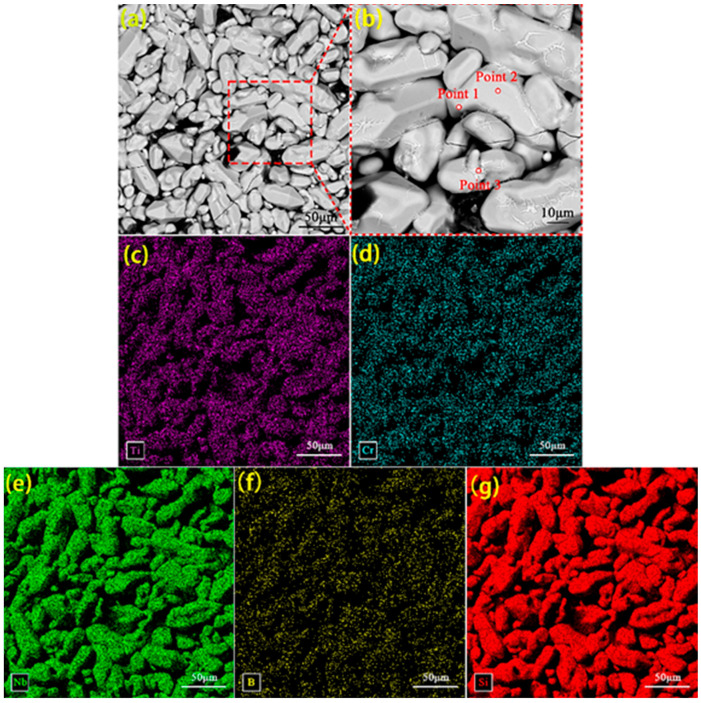
Image of EDS surface scan of the original coating: (**a**) surface image of the original coating; (**b**) enlarged image of the area shown in (**a**); elemental distribution of (**c**) Ti, (**d**) Cr, (**e**) Nb, (**f**) B, and (**g**) Si on the surface.

Figure 3 shows the surface EDS scan of the original coating surface. The EDS point scan results of each micro-region shown in Figure 3b are given in Table 1. Figure 3 indicates the presence of elements such as Ti, Cr, and B on the coating surface. Combined with the point scan results given in Table 1, Si and Nb can be seen to be the main components of the coating surface from Figure 3 with the ratio of (Nb + Cr) to Si ≈ 1:2. Therefore, NbSi_2_ should be the main component of the coating surface, with CrSi_2_ being the minor part. TiB_2_ is responsible for the element Ti (TiB_2_ is a modified additive during coating sintering and does not participate in the reaction during vacuum sintering), so a small amount of TiB_2_ is also present in the coating surface.

### 3.2. The Cross-Sectional Morphology and Mechanism of Formation of the Original Coating

**Figure 4:**
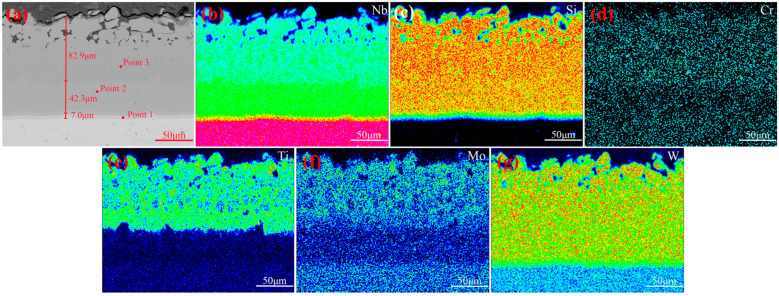
(**a**) Cross-sectional morphology of original coating; surface scanning results of (**b**) Nb; (**c**) Si; (**d**) Cr; (**e**) Ti; (**f**) Mo; (**g**) W.

The cross-sectional morphology, along with surface scanning results of the original coating, can be seen in Figure 4. A dense and overall uniform coating, with only a small number of pores, can be inferred from Figure 4a, which can be attributed to the incorporation of trace amounts of CeO_2_ and uniform slurry. The coating consisted of a diffusion layer and a main layer, which was further divided into two layers. The thickness of the diffusion layer was 7.0 μm, while the thicknesses of the inner main layer and outer main layer were 42.3 μm and 82.9 μm, respectively. According to Figure 4b–g, the elements Nb, Si, Cr, and W were distributed throughout the entire coating, while the elements Mo and Ti were distributed in the outer main layer of the coating. Table 2 shows the point scan results of the micro-area in Figure 4a. According to Table 2, during the sintering process, the elements Mo and Zr from the niobium alloy substrate diffused into the coating, and the amount of diffusion decreased with increasing distance from the substrate. However, for the element Mo, the content in the outer main layer is higher than that in the diffusion layer due to the addition of Mo during the second slurry sintering process, which is mainly distributed in the outer main layer. The content of W gradually increased toward the interior, as it was added during both sintering processes. Ti, Cr, and a small amount of Ce were distributed in the main layer of the coating, with their contents in the outer main layer being higher than those in the inner main layer. The addition of the Ce element is beneficial for promoting grain refinement and the densification of the coating [2]. The ratio of Nb to Si content in the diffusion layer was about 5:3, and it also contained small amounts of Mo and W, inferring that Nb_5_Si_3_ and low silicides of Mo/W were its main phases. The elemental ratio of (Nb, W, Cr, Mo):Si in the main coating was found to be 1:2, implying that NbSi_2_ and high silicides of Mo/W/Cr were its main phases. Moreover, a small amount of Ti, mainly existing in the form of TiB_2_, was also present in the outer main layer.

Based on the above conclusions and literature reports [19,22,30,31], it can be inferred that the following reactions primarily occur in the coating during the two-step slurry sintering process.

3Si(l) + 5Nb(s) → Nb_5_Si_3_(s) (1)

7Si(l) + Nb_5_Si_3_(s) → 5NbSi_2_(s) (2)

Si(l) + Nb(s) → NbSi_2_(s) (3)

2Si(l) + Mo(s) → MoSi_2_(s) (4)

2Si(l) + Cr(s) → CrSi_2_(s) (5)

2Si(l) + W(s) → WSi_2_(s) (6)

According to HSC calculations, the Gibbs free energies of reactions (1), (2), (3), (4), (5), and (6) at 1450 °C were −42.041 kcal/mol, −1.204 kcal/mol, −13.455 kcal/mol, −14.931 kcal/mol, −10.481 kcal/mol, and −9.305 kcal/mol, respectively. This indicates that all of the above reactions can take place spontaneously at 1450 °C. With Nb and Si as the main components of the slurry and reaction (1) having the minimum Gibbs free energy during the sintering process, Nb_5_Si_3_ (diffusion layer) is generated first, followed by reaction (2) to generate NbSi_2_ (main layer) as sintering proceeds. Other components present in the coating, such as Mo, W, and Cr, also react with Si in the main layer. Based on the Gibbs free energy comparison, the order of generation is as follows: MoSi_2_, CrSi_2_, and WSi_2_. Therefore, the coating structure was as follows: diffusion layer (Nb_5_Si_3_/low silicides of Mo and W) + inner main layer ((Nb,W,Mo,Cr)Si_2_) + outer main layer ((Nb,W,Mo,Cr)Si_2_/TiB_2_).

### 3.3. High-Temperature Oxidation Behavior of Coatings

The static oxidation life of the coating exceeded 20 h at 1700 °C, and morphology analysis of the coatings was carried out after varying oxidation times of 0.5 h, 1 h, 5 h, 10 h, 15 h, and 20 h.

**Figure 5:**
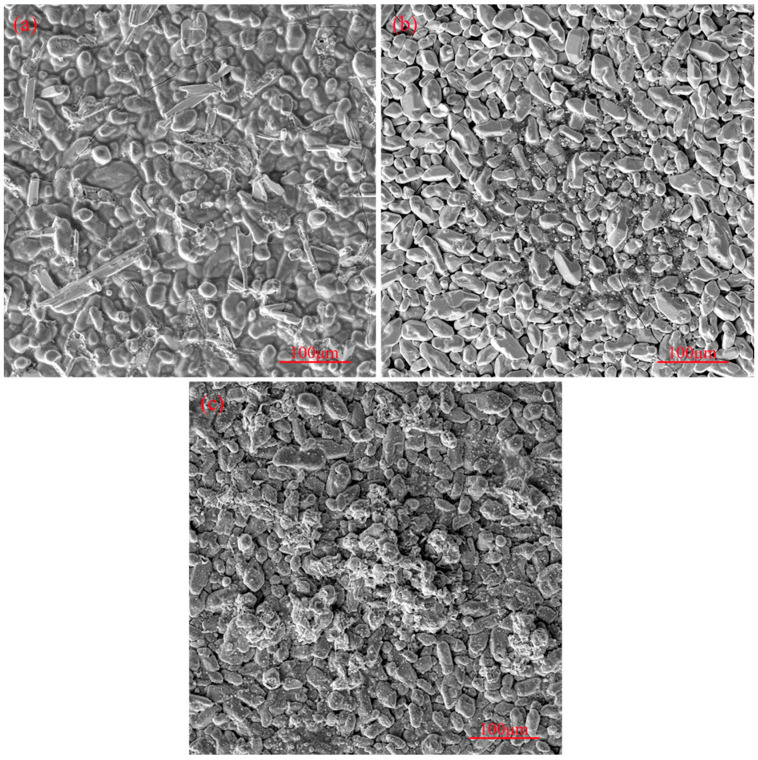
Surface morphology of oxide coating after (**a**) 1 h oxidation, (**b**) 10 h oxidation, and (**c**) 20 h oxidation.

The surface morphology of the coating after 1 h, 10 h, and 20 h of oxidation is shown in Figure 5. After each oxidation stage, a small number of non-film-forming agglomerates, which increase with increasing oxidation time, can be seen on the surface, as indicated in Figure 5. A small number of tiny cracks started to appear on the coating surface from 1 h, as seen in Figure 5a, but the cracks did not increase significantly during the subsequent oxidation process. After an oxidation period of 10 h, some small particles started to appear on the coating surface, as can be seen in Figure 5b. As shown in Figure 5c, after an oxidation time of 20 h, more particles appeared on the coating surface, and part of the coating structure exhibited oxidization and damage.

**Figure 6:**
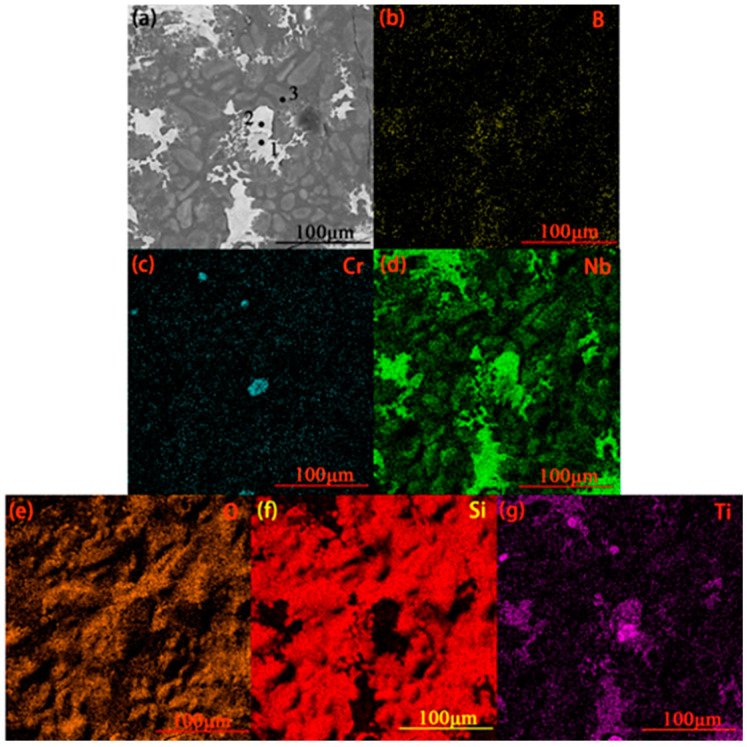
Scanning image of coating surface after 1 h of oxidation: (**a**) coating surface image; elemental scanning images of (**b**) B, (**c**) Cr, (**d**) Nb, (**e**) O, (**f**) Si, and (**g**) Ti.

Figure 6 shows the surface scan of the coating after 1 h of oxidation, and the point scan results of the micro-area shown in Figure 6a are presented in Table 3. According to Figure 6d,f, the white part of the surface after oxidation of the coating primarily contains the elements Nb and Ti, while the black part is constituted mainly by the element Si. According to Figure 6c, only a very small area on the coating surface is concentrated with Cr, thus indicating an extremely small distribution of Cr. Combining the surface scan results of elemental oxygen and the point scan results given in Table 3, the main components of the white part can be seen to be Nb_2_O_5_ and TiO_2_, along with a small amount of SiO_2_ and a very small amount of Cr_2_O_3_. The main component of the black part is SiO_2_, with a small amount of Nb_2_O_5_.

**Figure 7:**
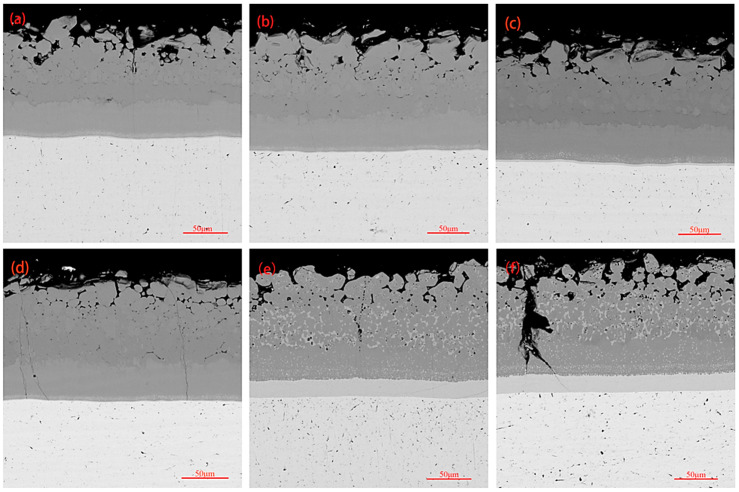
Cross-sectional morphologies of the coating after oxidation for different time intervals: (**a**) 0.5 h; (**b**) 1 h; (**c**) 5 h; (**d**) 10 h; (**e**) 15 h; (**f**) 20 h.

Cross-sectional morphologies of the coating after oxidation for 0.5 h, 1 h, 5 h, 10 h, 15 h, and 20 h are shown in Figure 7, showing that the cross-sectional morphology of the coatings up to 10 h of oxidation is similar to the cross-sectional morphology of the original coating, barring the appearance of a small number of microcracks on the cross-section of the coatings after 10 h of oxidation. Cross-sectional morphologies of the coatings after oxidation times of 15 h and 20 h were in the same form. A significant increase in the diffusion layer thickness of the coatings took place, and many white dispersed phases containing low silicides appeared on the main layer. Large cracks penetrating the entire main layer were observed in the coating after 20 h of oxidation. As the oxidation continued, oxygen penetrated the diffusion layer through the large cracks, leading to oxidation reactions. Due to the diffusion layer being primarily composed of low silicides, it quickly reacted with oxygen to cause cracks that rapidly extended to the substrate, resulting in coating failure. The appearance of large cracks throughout the entire coating indicated that the coating was about to reach its testing limit.

**Figure 8:**
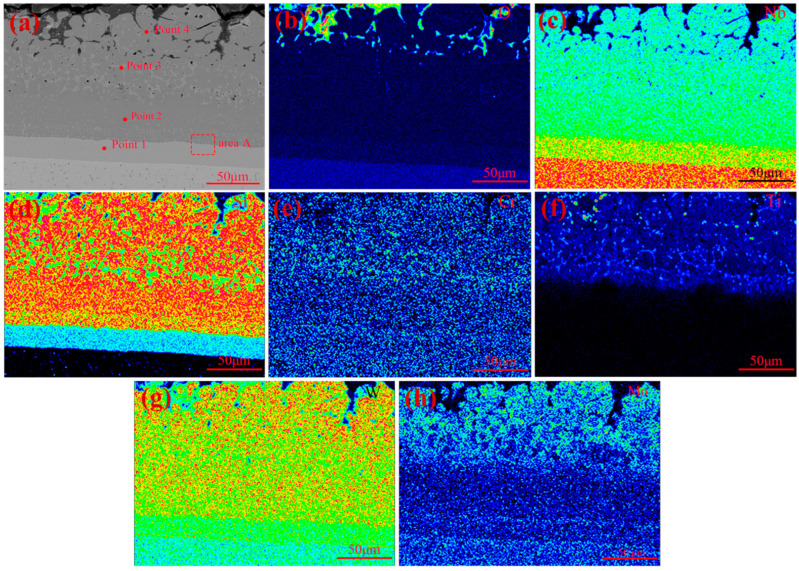
(**a**) Cross-sectional view of the coating after 10 h of oxidation; elemental mapping of (**b**) O, (**c**) Nb, (**d**) Si, (**e**) Cr, (**f**) Ti, (**g**) W, and (**h**) Mo in the surface of the coating after 10 h of oxidation.

The surface scan results of the coating after 10 h of oxidation are shown in Figure 8, with point scan results of the micro-area in Figure 8a being shown in Table 4. As per Figure 8b and the O content in each micro-area shown in Table 4, the O element mainly exists on the surface of the coating after 10 h of oxidation, with its content inside the coating being negligible. This indicates that the coating has a high density, effectively preventing inward penetration of oxygen. As indicated in Figure 8c,d and the Nb and Si contents in each micro-area shown in Table 4, the structure of the coating is still divided into the main layer and diffusion layer. The ratio of Nb to Si content in the main layer and the diffusion layer was about 1:2 and 5:3, respectively. Therefore, NbSi_2_ remains the main phase of the coating, while Nb_5_Si_3_ is the main phase of the diffusion layer. However, it is worth noting that more white dispersed phases can be seen in the outer main layer. The ratio of Nb to Si content in the white dispersed phase was between 1:2 and 5:3, indicating NbSi_2_ and Nb_5_Si_3_ to be the main components of the white dispersed phase in the main layer. The transformation from NbSi_2_ to Nb_5_Si_3_ occurs over an oxidation period of 10 h. According to Figure 8e–h and the content of modified elements in each micro-area shown in Table 4, it can be seen that Mo is almost absent in the coating. This may be due to the generation of MoO_3_ from the reaction between MoSi_2_ and oxygen, which evaporates at 1700 °C. The contents of Ti and Cr in the white dispersed phase of the outer main layer are much higher than those in other parts of the coating, and the W content in the coating remains basically unchanged before and after oxidation.

Based on the above analysis and surface scan results of the coating after 1 h of oxidation shown in Figure 6, as well as the corresponding micro-area point scan results, it can be concluded that the following reactions take place in the coating during the oxidation process [2,32,33,34]:

5NbSi_2_(s) + 7O_2_(g)→Nb_5_Si_3_(s) + 7SiO_2_(l) (7)

4Nb_5_Si_3_(s) + 37O_2_(g)→12SiO_2_(l) + 10Nb_2_O_5_(s) (8)

4CrSi_2_(s) + 11O_2_(g)→2Cr_2_O_3_(s) + 8SiO_2_(l) (9)

2TiB_2_(s) + 5O_2_(g)→2TiO_2_(s) + 2B_2_O_3_(l) (10)

2MoSi_2_ + 7O_2_(g)→2MoO_3_(g) + 4SiO_2_(l) (11)

WSi_2_ + 3.5O_2_(g)→WO_3_(g) + 2SiO_2_(l) (12)

According to HSC calculations, the Gibbs free energies for reactions (7), (8), (9), (10), (11), and (12) at 1700 °C are −133.83 kcal/mol, −99.60 kcal/mol, −116.08 kcal/mol, −111.37 kcal/mol, −84.69 kcal/mol, and −84.24 kcal/mol, respectively. These values indicate the spontaneity of the above reactions at 1700 °C.

Though the Gibbs free energy of the reaction between TiB_2_ and oxygen was not the lowest, it nevertheless comes into contact with oxygen first because it is located in the outer main layer of the coating. TiO_2_ is generated in the reaction, which covers the surface of the coating, and, due to its low melting point of 450 °C, B_2_O_3_ quickly evaporates into the atmosphere. Subsequently, Nb_5_Si_3_, Cr_2_O_3_, and Nb_2_O_5_ are generated due to the successive occurrence of reactions (7), (9), and (8). At the same time, the MoO_3_ and WO_3_ generated from reactions (11) and (12) continuously evaporate into the atmosphere. Subsequently, the reaction is accelerated due to the consumption of the product. Oxidation of the coating at high temperatures generates a semi-molten state of SiO_2_, which has a certain self-healing ability at 1700 °C and can repair surface defects of the coating, but SiO_2_ also has a certain level of volatility at 1700 °C. However, the added elements W and Mo preferentially oxidize to generate WO_3_ and MoO_3_, which, through volatilization, reduce the consumption of Si and volatilization of SiO_2_, thus further improving the performance of the coating [29,35]. The TiO_2_ generated during the oxidation process can synergistically prevent the inward diffusion of oxygen due to its high melting point and lower oxygen permeability compared with SiO_2_ [36].

As indicated by the above discussion, Table 3, and Figure 7, during the oxidation process of the coating, the NbSi_2_ in the main layer gradually transformed into Nb_5_Si_3_, which caused the diffusion layer thickness to gradually increase and the main layer thickness to decrease. The TiB_2_ and CrSi_2_ in the main layer were transformed into Cr_2_O_3_ and TiO_2_ during the oxidation process, which worked together with SiO_2_ to hinder the inward diffusion of oxygen. Moreover, MoSi_2_ and WSi_2_ in the main layer were transformed into low silicides during the oxidation process and then converted into WO_3_ and MoO_3_, which evaporated into the atmosphere. The transformation of the coating structure during the oxidation process primarily occurred from high silicide to low silicide and then to the corresponding oxide.

**Figure 9:**
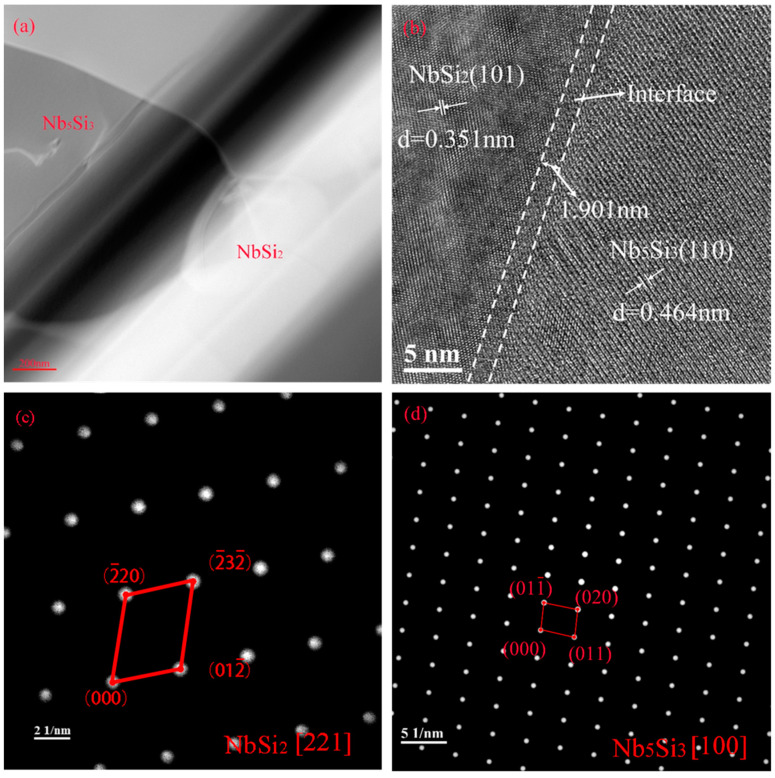
Microscopic morphology, high-resolution transmission image, and electron diffraction pattern of area A in Figure 8a: (**a**) TEM morphology; (**b**) HRTEM images; (**c**) electron diffraction pattern of NbSi_2_; (**d**) electron diffraction pattern of Nb_5_Si_3_.

The microstructure, high-resolution transmission image, and electron diffraction pattern of area A in Figure 8a are presented in Figure 9. As shown in Figure 9a,b, NbSi_2_ is the main phase of the main layer and Nb_5_Si_3_ is the main phase of the diffusion layer. The lattice spacings of the NbSi_2_ phase and Nb_5_Si_3_ phase are 0.351 nm and 0.464 nm, respectively. Grains of the two phases are interwoven and form an interface, with a thickness of approximately 1.901 nm. Further analysis of the electron diffraction patterns in Figure 9c,d confirms that the main layer and diffusion layer are NbSi_2_ and Nb_5_Si_3_ phases, respectively.

### 3.4. Thermal Shock Resistance of Coating

The results from the high-temperature thermal shock resistance test of the coating showed that, even after 1600 thermal shocks, the coating did not fail. An et al. [37] prepared a Si-Ti-Cr silicide coating on a Nb-Hf alloy surface, which could withstand 200 thermal shock cycles in an atmospheric environment ranging from room temperature to 1700 °C, indicating its excellent thermal shock resistance. The coating in this study withstood 1600 thermal shock cycles without failure in an atmospheric environment ranging from room temperature to 1700 °C, demonstrating its superior thermal shock performance and longer service life.

**Figure 10:**
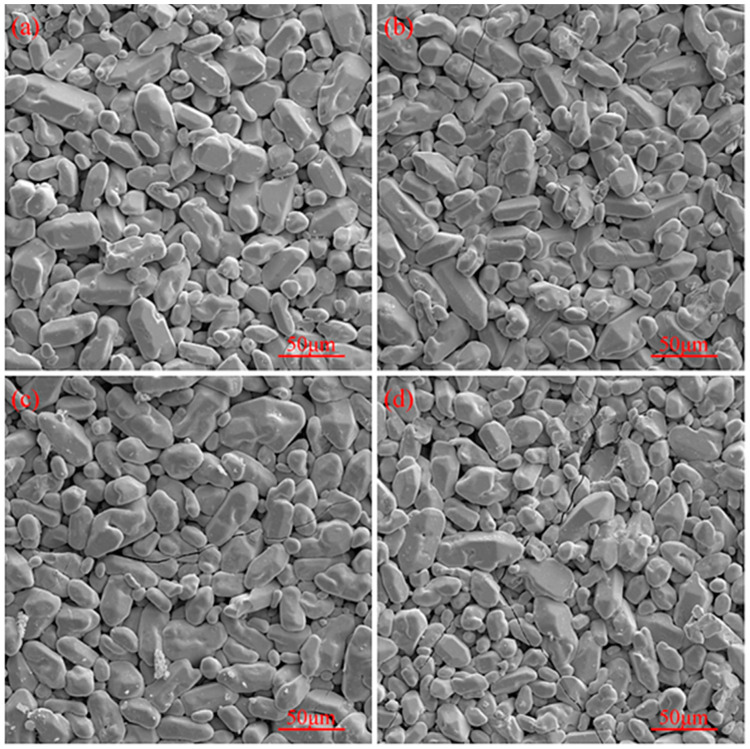
Surface morphology of the coating after high-temperature thermal shock resistance testing: (**a**) 400 cycles, (**b**) 800 cycles, (**c**) 1200 cycles, and (**d**) 1600 cycles of thermal shocks.

The surface morphologies of the coating after different numbers of high-temperature thermal shocks are shown in Figure 10. As evident from Figure 10, there were basically no cracks on the coating surface after 400 thermal shocks, and small cracks started to appear on the coating surface after 800 thermal shocks. With an increasing number of thermal shocks, more cracks started to appear on the coating surface, and the crack width also increased. After 1600 thermal shocks, larger cracks appeared on the coating surface. During the thermal shock process, Nb_2_O_5_ is formed by the reaction of NbSi_2_ on the coating surface with oxygen at high temperatures, and an increasing amount of Nb_2_O_5_ on the coating surface reduces the coating’s protective ability.

**Figure 11:**
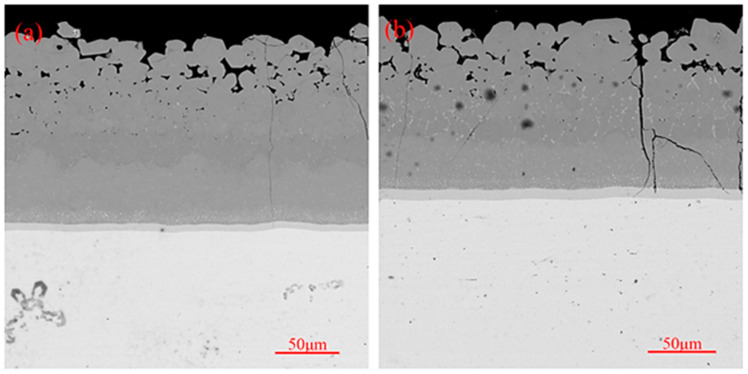

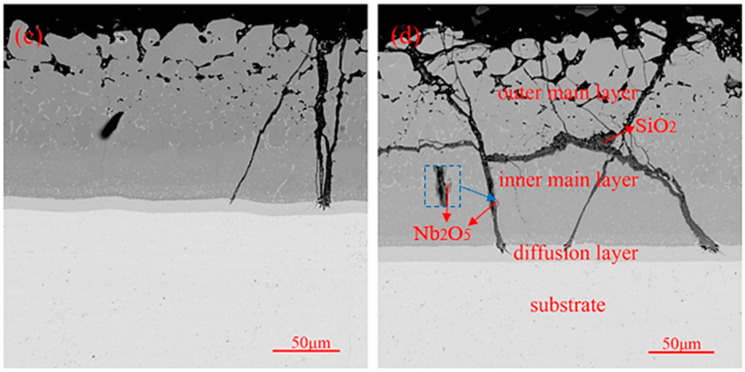
The cross-sectional morphologies of the coating after a varying number of cycles of high-temperature thermal shock resistance testing: (**a**) 400 cycles; (**b**) 800 cycles; (**c**) 1200 cycles; (**d**) 1600 cycles.

The coating shows excellent thermal shock resistance performance when there is good matching of thermal expansion coefficients between the coating layers. The thermal expansion coefficient of NbSi_2_ present in the main layer is 11.7 × 10^−6^/K, while the thermal expansion coefficient of Nb_5_Si_3_ present in the diffusion layer is 8.5 × 10^−6^/K, thus indicating a significant difference in the thermal expansion coefficients between the main layer and diffusion layer [38]. The introduction of CrSi_2_ (thermal expansion coefficient of 1.205 × 10^−5^/K) [39], WSi_2_ (thermal expansion coefficient of 8.5 × 10^−6^/K) [40], and MoSi_2_ (thermal expansion coefficient of 8.1 × 10^−5^/K) [38] can improve the thermal shock resistance of the coating by adjusting the difference in the thermal expansion coefficient between the main layer and the diffusion layer. The cross-sectional morphologies of the coating after the high-temperature thermal shock test can be seen in Figure 11. According to Figure 11a, only a small number of fine cracks seem to appear on the outer layer of the coating after 400 thermal shocks, while the inner layer of the coating remains dense and intact. After 800 thermal shocks, the appearance of a small number of longitudinal cracks penetrating both the inner and outer layers of the coating (Figure 11b) was observed. With a gradual increase in the number of thermal shocks to 1200, the cracks gradually increased (Figure 11c). After 1600 thermal shocks, transverse cracks were also observed inside the coating, in addition to longitudinal cracks penetrating the coating. However, in the absence of peeling or cracking of the substrate, the coating still indicates a certain protective ability. The thermal shock stress exceeding the fracture strength of the coating is the reason behind the appearance of cracks. In this instance, penetration and reaction of oxygen with NbSi_2_, CrSi_2_, and MoSi_2_ in the coating generates oxides such as SiO_2_. The main component inside the crack in Figure 11d after component analysis was found to be SiO_2_, along with a minor amount of Nb_2_O_5_. At 1700 °C, SiO_2_ is in a molten state with a certain fluidity, which can gradually repair cracks and prevent oxygen elements from entering the substrate through the fast diffusion channel of cracks. During the thermal shock process, phase transformation in the coating is similar to the oxidation process (mainly NbSi_2_), and, with an increasing number of thermal shocks, the diffusion layer thickens (with a gradual increase in Nb_5_Si_3_ in the diffusion layer). The difference between high-temperature thermal shock and high-temperature oxidation is that thermal shock can cause cyclic changes in the coating temperature, resulting in corresponding thermal stress, making it easier for internal cracks to occur in the coating and for the coating to fail. After 800 thermal shocks of the coating, a white dispersed low-silicon phase can be observed in the main layer, indicating the diffusion of Si and consumption of high silicides during the thermal shock process. At this point, cracks start to appear inside the coating, which further consume the silicon present in the coating. Although the composite oxide with SiO_2_ as the main body can delay further oxidation, the thermal expansion coefficient of 0.92 × 10^−6^/K is significantly different in comparison with that of 8–9 × 10^−6^/K for the coating main body. The stress concentration phenomenon is most prominent in the coating crack area during the rapid thermal shock process from room temperature to 1700 °C, leading to the continuous expansion of cracks. Internal cracks of the coating fuse under continuous stress superposition and phase change stress due to the expansion of the cracks and increase in density. This leads to the formation of transverse cracks, which may ultimately lead to failure and to the peeling of the coating during subsequent thermal shock processes [41,42].

**Figure 12:**
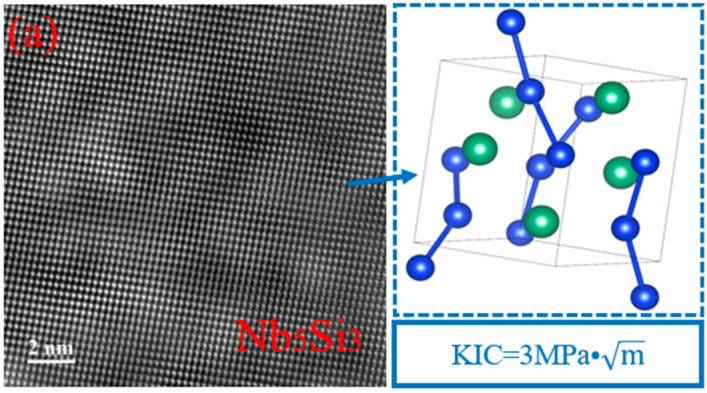

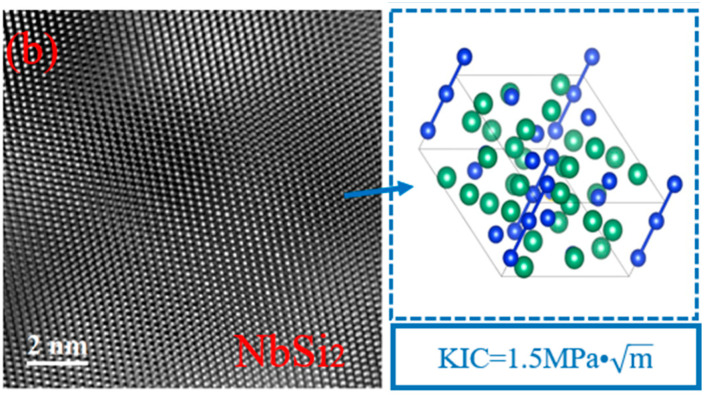
High-resolution transmission images, crystal structure diagrams, and fracture toughness values of Nb_5_Si_3_ and NbSi_2_: (**a**) Nb_5_Si_3_; (**b**) NbSi_2_.

The generation of transverse/longitudinal cracks during thermal shock is not only related to the stress concentration generated during thermal shock, but the fracture toughness of the main layer (NbSi_2_) and diffusion layer (Nb_5_Si_3_) are also influencing parameters. As shown in Figure 12, the crystal structure of Nb_5_Si_3_ in the diffusion layer is I4/mcm, with the room temperature fracture toughness being 3 MPa·$\sqrt{m}$ [43]. The crystal structure of NbSi_2_ in the main layer is P6222, and the room temperature fracture toughness is 1.5 MPa·$\sqrt{m}$ [44,45]. These all have a lower fracture toughness, and cracks start to occur when a certain degree of stress concentration occurs in the coating. However, improvement of the fracture toughness by MoSi_2_ and WSi_2_ in the main layer results in a very high overall thermal shock resistance of the coating [46,47], and the fracture toughness of the diffusion layer can also be improved by the composite structure formed by the Nb_5_Si_3_ and Nb alloy substrate [48]. Therefore, the coating can withstand higher stress, greatly improving its thermal shock resistance. As shown in Figure 11, during the thermal shock process, cracks are initiated from the main layer to terminate at the diffusion layer, which may also be related to the higher fracture toughness of the diffusion layer compared with the main layer.

## 4. Conclusions

In this study, two-step slurry sintering and halide-activated pack cementation were employed to prepare a novel TiB_2_-CeO_2_-modified (Nb,Mo,Cr,W)Si_2_ coating on the surface of Nb521. The surface morphology, cross-sectional morphology, microstructure, oxidation resistance, and thermal shock resistance of the coating were investigated. The results indicate that the developed coating exhibited excellent high-temperature oxidation resistance and thermal shock resistance, demonstrated by a protection life of over 20 h at 1700 °C and a thermal shock performance of over 1600 cycles from room temperature to 1700 °C. It is worth noting that, after oxidation at 1700 °C for 20 h, the coating exhibited large cracks that penetrated the main layer, and the coating then rapidly failed.

In a high-temperature oxidation environment, a TiO_2_-SiO_2_-Cr_2_O_3_ composite oxide film was formed on the coating surface due to the preferential reaction of TiB_2_ and (Mo,Cr)Si_2_ inside the coating with oxygen. This film, characterized by low oxygen permeability, effectively prevented the inward penetration of oxygen elements. The inward penetration of oxygen was further prevented by the dense structure of the composite coating. With continued high-temperature oxidation, the NbSi_2_ in the main layer of the coating gradually transformed into Nb_5_Si_3_, thus gradually increasing the diffusion layer. The presence of (Mo,Cr,W)Si_2_ inside the coating not only reduced the thermal stress generated by thermal shock cycles by adjusting the difference in thermal expansion coefficients between the layers of the composite coating but also improved the overall fracture toughness of the coating, thereby enhancing its thermal shock resistance.

## Figures and Tables

**Table 1:** EDS point scan results of the micro-area in Figure 3b.

Position	Composition (at%)				Main Phase
	Si	Ti	Cr	Nb	
1	63.54	11.26	0.52	24.68	(Nb,Cr)Si_2_, TiB_2_
2	68.89	5.22	0.76	25.13	(Nb,Cr)Si_2_, TiB_2_
3	62.57	6.82	1.18	29.43	(Nb,Cr)Si_2_, TiB_2_

**Table 2:** EPMA point scan results for micro-area in Figure 4a.

Position	Composition (at%)									Main Phase
	O	W	Cr	Si	Ti	Mo	Nb	Zr	Ce	
1	0.0000	2.9718	0.0000	36.6623	0.0000	1.1640	59.1116	0.0903	0.0000	Nb_5_Si_3_
2	0.0507	3.3061	0.1444	64.7589	0.1950	0.6390	30.8387	0.0360	0.0312	NbSi_2_
3	0.0885	3.8694	0.5652	64.2653	2.6175	1.4162	27.1306	0.0000	0.0473	NbSi_2_, TiB_2_

**Table 3:** Point scan results of micro-area in Figure 6a.

Position	Composition (at%)					Main Phase
	O	Si	Ti	Cr	Nb	
1	62.41	4.19	9.62	5.23	18.55	Nb_2_O_5_, Cr_2_O_3_, SiO_2_, TiO_2_
2	60.14	4.15	6.80	0.06	28.85	Nb_2_O_5_, TiO_2_, SiO_2_
3	67.21	28.28	0.35	0.29	3.86	SiO_2_, Nb_2_O_5_

**Table 4:** Point scan results of the micro-area in Figure 8a.

Position	Composition (at%)									Main Phase
	O	W	Nb	Si	Ti	Ce	Cr	Mo	Zr	
1	0.0000	2.9893	60.9344	35.9445	0.0000	0.0000	0.1137	0.0181	0.0000	Nb_5_Si_3_
2	0.0121	3.9550	31.7541	63.7609	0.4726	0.0065	0.0311	0.0000	0.0077	NbSi_2_
3	0.0538	3.7226	36.5971	44.9300	10.1253	0.0298	4.5414	0.0000	0.0000	Nb_5_Si_3_, TiB_2_
4	0.0822	3.8263	29.6296	63.4388	2.7873	0.0412	0.1874	0.0072	0.0000	NbSi_2_

## Data Availability

The data presented in this study are available upon request from the corresponding author due to technical confidentiality.
